# Evaluation of left atrial mechanical function and atrial conduction abnormalities in Maras powder (smokeless tobacco) users and smokers

**DOI:** 10.5830/CVJA-2014-070

**Published:** 2015

**Authors:** Ahmet Akcay, M Naci Aydin, Gurkan Acar, Mehmet Akgungor, Eren Cabioglu, İdris Ardic, Bulent Mese, Orhan Bozoglan, Mustafa Çetin, Musa Çakıcı

**Affiliations:** Department of Cardiology, Faculty of Medicine, Kahramanmaras Sutcuimam University, Kahramanmaras, Turkey; Department of Cardiology, Faculty of Medicine, Kahramanmaras Sutcuimam University, Kahramanmaras, Turkey; Department of Cardiology, Faculty of Medicine, Kahramanmaras Sutcuimam University, Kahramanmaras, Turkey; Department of Cardiology, Faculty of Medicine, Kahramanmaras Sutcuimam University, Kahramanmaras, Turkey; Department of Cardiology, Faculty of Medicine, Kahramanmaras Sutcuimam University, Kahramanmaras, Turkey; Department of Cardiology, Faculty of Medicine, Kahramanmaras Sutcuimam University, Kahramanmaras, Turkey; Department of Cardiovascular Surgery, Faculty of Medicine, Kahramanmaras Sutcu Imam University, Kahramanmaras, Turkey; Department of Cardiovascular Surgery, Faculty of Medicine, Kahramanmaras Sutcu Imam University, Kahramanmaras, Turkey; Department of Cardiology, Faculty of Medicine, Adıyaman University, Adıyaman, Turkey; Department of Cardiology, Faculty of Medicine, Adıyaman University, Adıyaman, Turkey

**Keywords:** smokeless tobacco, atrial electromechanical intervals, left atrial function

## Abstract

**Objective:**

In Turkey, a type of smokeless tobacco called Maras powder (MP) is widely used in the south-eastern region. Smokeless tobacco is found in preparations for chewing and for absorption by the nasal and oral mucosae. The purpose of this study was to investigate whether MP damages intra- and inter-atrial conduction delay and left atrial (LA) mechanical function as much as cigarette smoking.

**Method:**

A total of 150 chronic MP users (50 males, 32.5 ± 5.4 years), smokers (50 males, 32.1 ± 6.0 years) and controls (50 males, 30.1 ± 5.8 years) were included in the study. LA volumes were measured echocardiographically according to the biplane area–length method. Atrial electromechanical coupling was measured with tissue Doppler imaging and LA mechanical function parameters were calculated.

**Results:**

The LA passive emptying fraction was significantly decreased and LA active emptying volume (LAAEV) was significantly increased in the MP group (*p* = 0.012 and *p* = 0.024, respectively), and the LA active emptying fraction (LAAEF) was significantly increased in the smokers (*p* = 0.003). There was a positive correlation between the amount of MP used and smoking (pack years) with LAAEV and LAAEF (*r* = 0.26, *p* = 0.009 and *r* = 0.25, *p* = 0.013, respectively). Lateral atrial electromechanical intervals (PA) were significantly higher in MP users, and the septal mitral PA was statistically higher in the smokers (*p* = 0.05 and *p* = 0.04, respectively).

**Conclusion:**

We suggest that atrial electromechanical coupling intervals were prolonged and LA mechanical function was impaired in MP users and smokers, but there was no significant difference between the MP users and smokers. These findings may be markers of subclinical cardiac involvement and tendency for atrial fibrillation.

## Abstract

Tobacco use can be classified into smoking and smokeless tobacco. Smokeless tobacco is chewed or is absorbed by the nasal and oral mucosae. A type of smokeless tobacco called Maras powder (MP) is used mostly in the south-eastern region of Turkey, and in many cases users become addicted. It is obtained from a tobacco plant species known as Nicotiana rustica Linn. Nicotine concentrations in the tobacco used to produce MP are eight to 10 times higher than those in tobacco used to produce cigarettes.^1^ MP and its negative effects on the cardiovascular system have been well studied. MP is consumed in such a way that increase in oxidative stress is inevitable and as a result it accelerates the atherosclerotic process.^2^,^3^

Cigarette smoke includes nicotine and toxic substances such as carbon monoxide and polycyclic aromatic hydrocarbons.^4^ Inhalation of these substances predisposes to several different atherosclerotic syndromes,^5^,^6^ and is also associated with the occurrence of cardiac arrhythmia.^7^,^8^

The pathophysiological mechanism of cigarette smoking-induced cardiac arrhythmia is complicated, and the pro-fibrotic effect of nicotine on myocardial tissue with its consequent increased susceptibility to catecholamines, may play a role. Moreover, other components of cigarette smoking, such as carbon monoxide, as well as oxidative stress, are likely to cause the generation of arrhythmias. It is also known that cigarette smoking leads to cardiac autonomic dysfunction,^9^ and it has been implicated in prolonged QT intervals in healthy individuals.^10^ However, the nicotine concentration in the blood is more likely to cause the pro-arrhythmic effect of cigarette smoking.^7^,^11^ The risk of atrial and ventricular arrhythmia rises due to increased nicotine levels.^9^-^12^

The prolongation of intra- and inter-atrial electromechanical intervals and the inhomogeneous propagation of sinus impulses are well-known electrophysiological characteristics of atria that are prone to fibrillation.^13^ Left atrial (LA) volume and LA mechanical function have recently been identified as a potential indicator of cardiac disease and arrhythmias.^14^,^15^ Prolongation of atrial electromechanical interval and impaired LA mechanical function are associated with adverse clinical events, including atrial fibrillation, stroke, diastolic dysfunction and left ventricular failure.^16^,^17^

LA mechanical function and atrial conduction abnormalities have not been investigated in MP users and smokers. Therefore, our study was planned to evaluate whether MP damages intra- and inter-atrial conduction intervals and LA mechanical function as much as cigarette smoking.

## Methods

A total of 150 chronic MP users (50 males, mean age 32.5 ± 5.4 years), cigarette smokers (50 males, mean age 32.1 ± 6.0 years) and controls (50 males, mean age 30.1 ± 5.8 years) who referred to various out-patient departments (cardiology clinic, public health clinic, internal medicine clinic, cardiovascular surgery clinic) and were matched for age and gender, were included in the study. A medical history was taken and detailed physical examinations were performed on all subjects.

The inclusion criterion was using MP for at least three years. A package of MP was considered sufficient to provide use of the powder for 20 occasions. Duration and frequency of MP use, duration of cigarette smoking and number of cigarettes smoked throughout the day were recorded. The entire study population’s demographic characteristics, biochemical parameters, lipid values and ECGs were obtained.

Exclusion criteria were: history of coronary artery disease, arterial hypertension, hypercholesterolaemia, diabetes mellitus, primary cardiomyopathy, valvular heart disease, left ventricular ejection fraction (LVEF) less than 50%, bundle branch block, LV wall motion abnormality, renal failure, atrioventricular conduction abnormalities on electrocardiogram, thyroid dysfunction, anaemia, electrolyte imbalance, pulmonary disease, and poor-quality echocardiographic and electrocardiographic imaging.

All patients were in sinus rhythm, and none was taking medication such as anti-arrhythmics, antihistamines, tricyclic antidepressants and antipsychotics. Written informed consent was obtained from each subject. The institutional ethics committee approved the study protocol.

## Echocardiography

In this study, a Vingmed Vivid Seven Pro, Doppler echocardiographic (GE Ultrasound, Horten, Norway) unit with a 2–4 MHz FPA probe was used. Tissue Doppler (TDI) echocardiography was performed with a transducer frequency of 3.5–4.0 MHz, adjusting the spectral pulsed Doppler signal filters to obtain the Nyquist limit of 15–20 cm/s, and using the minimal optimal gain setting. The monitor sweep speed was set at 50–100 mm/s to optimise the spectral display of myocardial velocities.

A 12-lead electrocardiogram recording obtained from the same derivation (DII derivation) was recorded continuously during the echocardiographic examination in all study subjects. Two-dimensional, M-mode, pulsed and colour-flow Doppler echocardiographic examinations were performed by a cardiologist who was blinded to the clinical details and findings of other examinations of each subject and control. During echocardiography, continuous one-lead electrocardiographic recordings were obtained. LA volumetric parameters were measured by transthoracic echocardiography in the left lateral position, in parasternal long axis, apical four chambers and two chambers. M-mode measurements and conventional Doppler echocardiographic examinations were performed according to the guidelines of the American Society of Echocardiography.^18^

All measurements were recorded as averages of three cardiac cycles. LA dimension, LV end-systolic and end-diastolic dimensions, diastolic ventricular septal thickness, and diastolic LV posterior wall thickness were measured in the parasternal long-axis view. LVEF was estimated using the Simpson’s rule. All echocardiographic examinations were performed by the same cardiologist.

LA volumes were measured echocardiographically using the biplane area–length method in apical four-chamber and two-chamber views. LA maximal volume (V_max_) was recorded at the onset of mitral opening, LA minimal volume (V_min_) was recorded at the onset of mitral closure, and LA pre-systolic volume (V_p_) was recorded at the beginning of the atrial systole (P wave on ECG). All volume measurements were corrected to body surface area, expressed as ml/m^2^ and the following LA emptying function parameters were calculated:^19^

LA passive emptying volume (LAPEV) = V_max_ – V_p_

LA passive emptying fraction (LAPEF) = LAPEV/V_max_

LA active emptying volume (LAAEV) = V_p_ – V_min_

LA active emptying fraction (LAAEF) = LAAEV/V_p_

LA total emptying volume (LATEV) = V_max_ – V_min_

LA total emptying fraction (LATEF) = LATEV/V_max_

All measurements were repeated during three consecutive heart beats and the average of three consecutive measurements was obtained.

## Atrial electromechanical coupling measurements

For atrial electromechanical intervals in the apical four-chamber view, the pulsed Doppler sample volume was placed at the level of the LV lateral mitral annulus, septal mitral annulus and right ventricular (RV) tricuspid annulus. Atrial electromechanical intervals (PA) were measured as the time interval between the onset of the P wave on the electrocardiogram and the beginning of the late diastolic A wave at the lateral mitral annulus (lateral PA), septal mitral annulus (septal PA), and RV tricuspid annulus (RV PA). The difference between lateral PA and RV PA (lateral PA–RV PA) was defined as inter-atrial dyssynchrony, and the difference between septal PA and RV PA (septal PA–RV PA) as intra-atrial dyssynchrony.^20^

## Reproducibility

Intra-observer variability was assessed in 20 subjects selected randomly from the study groups by repeating the measurements under the same basal conditions. Reproducibility of atrial electromechanical intervals obtained by TDI was assessed by coefficient of variation (CV) between measurements. Intra- and inter-observer coefficients of variation for echocardiographic measurements were found to be < 5% and non-significant.

## Statistical analysis

All analyses were conducted using SPSS 15.0 (SPSS for Windows 15.0, Chicago, IL, USA). Continuous variables were expressed as mean ± standard deviation; categorical variables were defined as percentages. Categorical data were compared with the chi-square test. All numerical variables of the study groups presented a normal distribution. Mean values of continuous variables were therefore compared using analysis of variance (ANOVA), and the *post hoc* Tukey test was used for comparison of groups. Pearson’s correlation coefficients were used to assess the strength of the relationship between continuous variables. A *p*-value < 0.05 was considered significant. We performed the power analysis using G*Power software version 3.1.5. The power of our study was calculated to be 0.96.

## Results

Basic clinical and laboratory values, and M-Mode and two-dimensional echocardiographic measurements of the three groups are listed in Table 1. Age, body mass index (BMI), body surface area (BSA), systolic and diastolic blood pressure, heart rate, LV end-diastolic diameter, interventricular septal thickness, posterior wall thickness, LVEF, LA dimension, mitral E velocity, mitral A velocity, E/A ratio, systolic pulmonary artery pressure, and glucose, total cholesterol, triglyceride, high-density lipoprotein (HDL) cholesterol and low-density lipoprotein (LDL) cholesterol levels were similar between the three groups (p > 0.05).

**Table 1:** Clinical characteristics, laboratory and echocardiographic findings of the groups

	*Group I controls (n = 50)*	*Group II smokers (n = 50)*	*Group III MP users (n = 50)*	*p-value*
Age (years)	30.1 ± 5.8	32.1 ± 6	32.5 ± 5.4	0.086
BMI (kg/m^2^)	26.3 ± 3.7	25.9 ± 3.5	26.9 ± 3.9	0.462
BSA (m^2^)	1.96 ± 0.14	1.95 ± 0.15	1.92 ± 0.16	0.388
Systolic BP (mmHg)	125.3 ± 7.4	121.2 ± 6.4	120.7 ± 8.3	0.354
Diastolic BP (mmHg)	79.5 ± 6.3	78.5 ± 5.4	77.9 ± 5.8	0.789
Heart rate (beats/min)	72.2 ± 10.4	74.6 ± 9.6	74.5 ± 10.1	0.407
LVEDD (mm)	48.7 ± 3.1	47.7 ± 2.5	48.6 ± 3.4	0.224
IVS	9.5 ± 0.8	9.9 ± 0.8	9.8 ± 0.8	0.060
PW	8.7 ± 0.7	8.9 ± 0.6	9.1 ± 0.7	0.087
LVEF (%)	69.8 ± 2.6	68.4 ± 3.2	68 ± 3.4	0.314
LA dimension (mm)	33.2 ± 3.1	32.9 ± 2.7	34.3 ± 3.1	0.071
Mitral E velocity (cm/s)	78.9 ± 14.5	78.5 ± 14.8	81.0 ± 16.7	0.429
Mitral A velocity (cm/s)	57.3 ± 12.9	56.7 ± 10.9	56.4 ± 10.3	0.922
E/A	1.44 ± 0.38	1.41 ± 0.28	1.46 ± 0.32	0.398
sPAP (mmHg)	19.5 ± 3.8	19.4 ± 3.9	21.1 ± 3.5	0.057
Glucose (mg/dl)	92.7 ± 15.7	92.8 ±16.6	93.7 ± 19.8	0.951
(mmol/l)	5.14 ± 0.87	5.15 ± 0.92	5.20 ± 1.10	
Total cholesterol (mg/dl)	184.5 ± 41.4	180.5 ± 46.8	174.0 ± 35	0.444
(mmol/l)	4.78 ± 1.07	4.67 ± 1.21	4.51 ± 0.91	
Triglycerides (mg/dl)	151 ± 88.2	173.7 ± 112.4	186.6 ± 130	0.275
(mmol/l)	1.71 ± 1.00	1.96 ± 1.27	2.11 ± 1.47	
HDL cholesterol (mg/dl)	42.8 ± 8.7	39.2 ± 9.1	39.8 ± 8.8	0.105
(mmol/l)	1.11 ± 0.23	1.02 ± 0.24	1.03 ± 0.23	
LDL cholesterol (mg/dl)	110.2 ± 34.6	106.9 ± 36.5	100 ± 27.7	0.297
(mmol/l)	2.85 ± 0.90	2.77 ± 0.95	2.59 ± 0.72	
Duration (pack years)	–	13.6 ± 6.2	10.9 ± 6.6	0.014*

BMI: body mass index, BSA: body surface area, BP: blood pressure, LV: left ventricular, LVEDD: LV end-diastolic dimension, EF: ejection fraction, HDL: high-density lipoprotein, LDL: low-density lipoprotein. All *p*-values > 0.05 (ANOVA test). *p-value for group II vs III.

## Left atrial mechanical function

**Figure 1.**
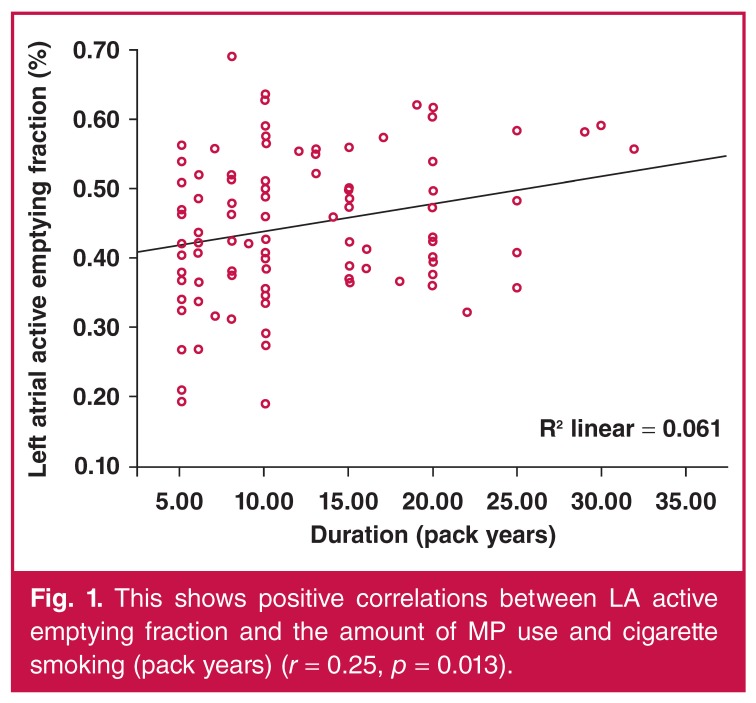
This shows positive correlations between LA active emptying fraction and the amount of MP use and cigarette smoking (pack years) (*r* = 0.25, *p* = 0.013).

**Figure 2.**
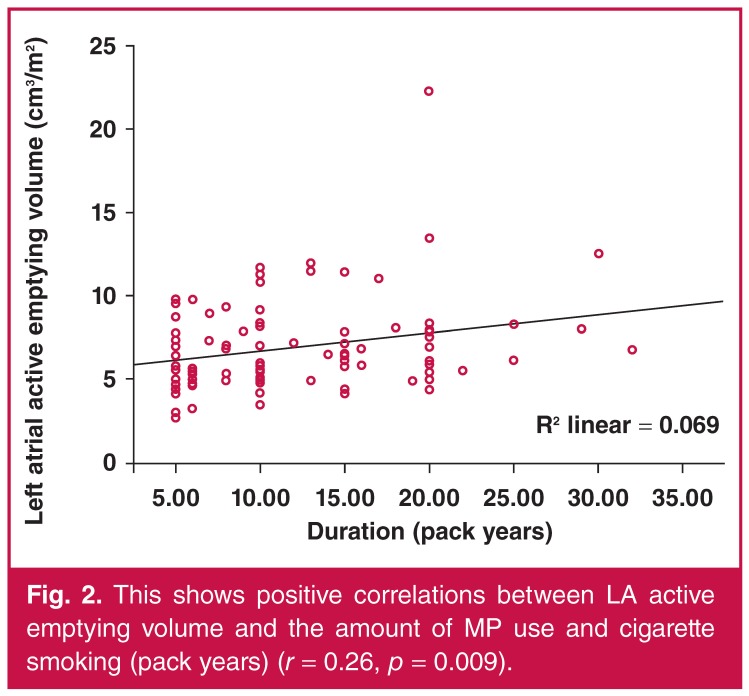
This shows positive correlations between LA active emptying volume and the amount of MP use and cigarette smoking (pack years) (*r* = 0.26, *p* = 0.009).

The three groups were similar with regard to V_max_, V_min_ and V_p_, LAPEV, LATEV and LATEF (*p* = 0.322, *p* = 0.052, *p* = 0.087, *p* = 0.161, *p* = 0.976, *p* = 0.170, respectively). However, LAPEF was significantly decreased and LAAEV was significantly increased in the MP groups but not in the control group (*p* = 0.012 and *p* = 0.024, respectively), and LAAEF was significantly increased in the cigarette smoking group and not in the control group (*p* = 0.003) (Table 2). The amount of MP used and cigarette smoking (pack years) were weakly but significantly correlated with LAAEV and LAAEF (*r* = 0.26, *p* = 0.009, *r* = 0.25, *p* = 0.013, respectively) (Figs 1, 2).

**Table 2:** Left atrial volume measurements in smokers, Maras powder users and control groups

	*Group I controls (n = 50)*	*Group II smokers (n = 50)*	*Group III MP users (n = 50)*	*p-value*
LA maximal volume (cm^3^/m^2^)	22.9 ± 5.3	21.7 ± 6.2	23.3 ± 5.5	0.322
LA minimal volume (cm^3^/m^2^)	8.9 ± 3.4	7.8 ± 2.8	9.2 ± 3.2	0.052
Volume at the onset of atrial systole (cm^3^/m^2^)	14.7 ± 4.7	14.5 ± 4.8	16.4 ± 4.3	0.087
LA passive emptying volume (cm^3^/m^2^)	8.2 ± 3.2	7.1 ± 3.4	6.9 ± 4.3	0.161
LA passive emptying fraction (%)	0.36 ± 0.11	0.32 ± 0.12	0.28 ± 0.14*	0.012
LA active emptying volume (cm^3^/m^2^)	5.8 ± 2.2	6.8 ± 3.0	7.2 ± 2.5#	0.024
LA active emptying fraction (%)	0.39 ± 0.10	0.46 ± 0.10§	0.43 ± 0.11	0.003
LA total emptying volume (cm^3^/m^2^)	14.0 ± 3.3	13.9 ± 4.6	14.1 ± 4.2	0.976
LA total emptying fraction (%)	0.61 ± 0.9	0.63 ± 0.9	0.6 ± 1.0	0.170

LA = left atrial. *p = 0.012 versus group I, ^#^*p* = 0.024 versus group I, ^#^*p* = 0.003 versus group I.

## Atrial electromechanical intervals

The atrial electromechanical coupling intervals measured from different sites by TDI are shown in Table 3. PA lateral was significantly higher in the MP users than in the controls. Also, PA septum was statistically higher in cigarette smokers than in the controls (*p* = 0.05 and *p* = 0.04, respectively). Intra- and inter-atrial dyssynchrony was prolonged in both MP users and cigarette smokers, but did not reach statistical significance. The measurements of atrial electromechanical coupling intervals were similar between MP users and cigarette smokers.

**Table 3:** Findings of atrial electromechanical coupling measured by tissue Doppler imaging

	*Group I controls (n = 50)*	*Group II smokers (n = 50)*	*Group III MP users (n = 50)*	*p-value*
Lateral PA (ms)	48.3 ± 9.8	53.9 ± 12.9	54.1 ± 14.1§	0.032
Septal PA (ms)	40.1 ± 8.7	46.3 ± 13.3#	45.8 ± 15.2	0.028
RV PA (ms)	34.1 ± 8.4	38.4 ± 9.9	37.5 ± 12.7	0.098
Lateral PA–septal PA (ms)	8.1 ± 3.5	7.9 ± 4.3	8.2 ± 3.9	0.687
Lateral PA–RV PA (ms)*	14.2 ± 8.9	15.5 ± 10.1	16.5 ± 8.4	0.443
Septal PA–RV PA (ms)**	6.0 ± 5.9	7.9 ± 8.8	8.2 ± 7.7	0.287

PA: the interval with tissue Doppler imaging, from the onset of P wave on the surface electrocardiogram to the beginning of the late diastolic wave (Am wave). *Inter-atrial dyssynchrony, **intra-atrial dyssynchrony. ^§^*p* = 0.05 versus group I, ^#^*p*sp = 0.04 versus group I.

## Discussion

The main finding of this study was that atrial electromechanical coupling intervals were prolonged and left atrial mechanical function was impaired in MP users and cigarette smokers compared to healthy controls, but there was no significant difference between the MP users and cigarette smokers. Also, the amount of MP used and cigarette smoking (pack years) was correlated with LAAEV and LAAEF. This is the first study to determine the atrial electromechanical and mechanical functions of MP users and cigarette smokers.

Cigarettes have widespread use and their smoke contains more than 4 000 toxic compounds, mainly nicotine. Various clinical and pathological investigations have shown that cigarette smoking caused atherosclerosis, myocardial infarction and heart failure,^4^ and nicotine abuse is associated with the occurrence of cardiac arrhythmias.^9^,^12^ The pro-arrhythmic effect of cigarette smoking seems to depend on the nicotine concentration in the blood.^9^ Increased nicotine levels increase atrial and ventricular vulnerability to fibrillation.^9^,^12^ These effects are more likely to depend on the inhibition of ion channels and conduction-slowing properties.

One factor known to cause a substantial slowing of electrical impulse propagation in cardiac tissue is an increase in the amount of interstitial collagen. It has been found that the prolonged administration of nicotine is also associated with the loss of intracellular K^+^ and the emergence of cardiac necrosis.^21^

Experimentally, Goette et al. established a linear correlation between nicotine dose and atrial collagen expression, leading to symptomatic atrial fibrosis at a younger age.^22^ In previous reports, atrial conduction time was found to be prolonged independently of LA dilatation.^20^,^23^ In a study consisting of 50 smokers and 40 non-smokers, it was found that inter- and intra-atrial electromechanical delay was significantly higher in cigarette smokers compared with non-smokers, and the amount of smoking was strongly correlated with inter-atrial electromechanical delay.^24^

Distinct from that study, our study contained a third group, the MP users, and we also investigated left atrial mechanical function in our study population. Likewise, we found that although LA was not dilated, atrial conduction time was prolonged in both MP users and smokers. This may be explained as the negative effects of nicotine on cardiac structure and function. The development of interstitial fibrosis affects chamber geometry and mechanical performance of the heart and enhances the likelihood of cardiac arrhythmias, such as atrial fibrillation (AF). Moreover, in prospective, population-based and 16-year follow-up studies, it has been shown that smoking was associated with incidence of AF, with more than a two-fold increased risk of AF attributed to current smoking.^25^ The risk of AF increased with increasing cigarette years of smoking, and appeared to be somewhat greater among current smokers than former smokers with similar cigarette years of smoking.^25^

MP is a form of smokeless tobacco. The ash in this mixture transforms the alkaloids into a base form and provides easy absorption from the buccal mucosa.^26^ As MP contains six- to10-fold more nicotine than cigarettes, it is preferred by addicts. It has been shown that urinary cotinine levels were three times higher in MP users than in cigarette smokers.^27^ Besides, MP is closely associated with traditional cardiovascular risk factors and endothelial dysfunction, as detected by low plasma NO levels.^3^ MP increases oxidative stress and lipid peroxidation levels, which are the best indicators of cytological damage.^28^

Because of the deleterious effects of cigarette smoking, especially the nicotine blood level, it stimulates the sympathetic nerve endings and increases adrenaline release, which in turn increases cardiovascular abnormalities.^29^ The probability of abnormalities of the cardiac conduction system and the occurrence of cardiac arrhythmias in MP users with a high blood nicotine level may also be high. Furthermore, it is well known that myocardial ischaemia, systemic inflammation, oxidative stress and increased sympathetic activity play an important role in the pathogenesis of atrial fibrillation (AF). In addition, a case report was found that indicated that the use of MP may lead to the occurrence of paroxysmal AF.^30^

Atrial electromechanical delay can be measured by invasive or non-invasive methods. Recent studies have assessed atrial electromechanical delay with TDI echocardiography, which is an alternative, non-invasive method to invasive electrophysiological studies.^31^,^32^ In previous studies, it was found that atrial conduction time measured by TDI was an independent predictor of new-onset or recurrence of AF.^13^,^31^

LA mechanical function consists of reservoir, and passive and active emptying functions at different stages of the cardiac cycle. The reservoir function takes effect during ventricular systole, the passive emptying function in early diastole and the active emptying function during ventricular diastole in the presence of sinus rhythm. When LV dysfunction develops, the LA may possibly preserve adequate cardiac output by regulation of the reservoir and booster pump function.

We demonstrated that LA mechanical function was impaired in MP users and smokers, but there was no significant difference between the MP users and cigarette smokers. LAPEF was significantly decreased and LAAEV was significantly increased in the MP group but not in the control group. LAAEF was significantly increased in the cigarette smoking group but not in the control group. LAPEF is related to elevated end-diastolic LV pressure, and the increase in LAAEV is associated with a compensatory mechanism in LA contraction. These findings may be an indirect indication that nicotine has greater effects on the atrial tissue over time.

Additionally, Eroglu *et al.* demonstrated that chronic cigarette smoking caused changes in long-axis systolic and diastolic function of the right and left ventricles in healthy young subjects.^33^ Our study population consisted of relatively young subjects. LA electromechanical and mechanical functions were impaired in our smokers and MP users without overt cardiovascular disease, probably due to the negative effects of nicotine. This may be an early sign of atrial dysfunction preceding AF in these subjects.

## Study limitations

The major limitation of our study was its cross-sectional design and lack of follow up of the patients. The subjects could not be followed up for episodes of arrhythmia, therefore, we do not know whether parameters of prolonged atrial electromechanical delay and impaired LA mechanical function can be used for the prediction of arrhythmias and heart failure in MP users and smokers. For these reasons, long-term follow up and large-scale prospective studies are needed to determine the predictive value of prolonged atrial electromechanical delay parameters and LA mechanical function in this population.

The absence of detailed parameters of diastolic function and measurement of blood nicotine levels were also potential limitations of our study. Furthermore, factors such as the subjects’ diet and exercise habits, which may affect diastolic function, could not be evaluated in our study.

## Conclusion

On the basis of our findings, we suggest that atrial electromechanical coupling intervals were prolonged and left atrial mechanical function was impaired in MP users and cigarette smokers, but there was no significant difference between MP users and cigarette smokers. Furthermore, the amount of MP use and cigarette smoking (pack years) was correlated with LAAEV and LAAEF. These findings may be markers of subclinical cardiac involvement and a risk for AF.
